# Reliability of Sternocleidomastoid Muscle Stiffness Assessment Using Shear-Wave Elastography Under a Standardized Protocol with Novice and Experienced Examiners: An Intra- and Inter-Examiner Reliability Study

**DOI:** 10.3390/medicina62020267

**Published:** 2026-01-27

**Authors:** Germán Monclús-Díez, Sandra Sánchez-Jorge, Jorge Buffet-García, Mónica López-Redondo, Davinia Vicente-Campos, Umut Varol, Ricardo Ortega-Santiago, Juan Antonio Valera-Calero

**Affiliations:** 1 Department of Physiotherapy, Faculty of Nursery, Physiotherapy and Podiatry, Complutense University of Madrid, 28040 Madrid, Spain; gmonclus@ucm.es; 2 Faculty of Health Sciences, Universidad Francisco de Vitoria, 28223 Madrid, Spain; j.buffet.prof@ufv.es (J.B.-G.); monica.lopezredondo@ufv.es (M.L.-R.); davinia.vicente@ufv.es (D.V.-C.); 3 Grupo de Investigación de Alto Rendimiento en Evaluación Multidimensional y Tratamiento del Dolor Crónico, Universidad Rey Juan Carlos, 28922 Alcorcón, Spainricardo.ortega@urjc.es (R.O.-S.); 4 Department of Physical Therapy, Occupational Therapy, Rehabilitation and Physical Medicine, Universidad Rey Juan Carlos, 28922 Alcorcón, Spain

**Keywords:** agreement, reproducibility, shear-wave elastography, sternocleidomastoid muscle, ultrasound imaging

## Abstract

*Background and Objectives*: Sternocleidomastoid (SCM) dysfunction is commonly implicated in several musculoskeletal conditions. Accordingly, shear-wave elastography has been used to characterize SCM stiffness in asymptomatic and clinical cohorts. However, the only reproducibility study available reported limited reliability, so clinical interpretations should be made with caution. Therefore, this study revisits key methodological aspects of that protocol to assess intra-examiner reliability and includes two examiners with different levels of expertise to evaluate inter-examiner reliability. *Materials and Methods*: A longitudinal observational study was conducted, recruiting twenty-five asymptomatic participants. Two examiners with different experience levels participated in this study after following structured training. For each side, images were obtained in immediate succession in the sequence experienced–novice–experienced–novice (with side order randomized), using an ROI spanning full muscle thickness, stabilizing approximately 10 s before freezing to record Young’s modulus and shear-wave speed. *Results*: Inter-examiner agreement was good–excellent: single-measurement ICCs were 0.77–0.86, improving to 0.79–0.87 when averaging two trials, which also reduced the standard error of measurement (SEM) and minimal detectable changes (MDCs). Between-examiner mean differences were small and nonsignificant (*p* ≥ 0.068). Intra-examiner reliability was excellent (ICC ≈ 0.93–0.94) with small absolute errors. Precision was high (SEM ~5–6 kPa; 0.22 m/s), and MDCs were ~15–16 kPa and ~0.60 m/s, with no trial-to-trial bias (all *p* ≥ 0.311). *Conclusions*: The revised protocol showed excellent intra-examiner repeatability and good–excellent inter-examiner reliability with minimal bias. Averaging two acquisitions improved precision, while a single operator optimized longitudinal stability.

## 1. Introduction

The sternocleidomastoid (SCM) lies along the front side of the neck and arises from two separate origins: a sternal slip from the manubrium and a clavicular slip from the medial third of the clavicle. These fibers join and attach to the mastoid process of the temporal bone and to the lateral segment of the superior nuchal line on the occiput. Motor supply is provided by the spinal accessory nerve (cranial nerve XI), while proprioceptive input comes from the cervical plexus (C2–C3) [1,2]. Acting on one side, the SCM laterally bends the neck to the same side and rotates the head to the opposite side; acting on both sides, it flexes the cervical spine. When the neck is fixed, it can elevate the sternum and clavicle, assisting with forced inspiration, underscoring its combined roles in cervicocephalic movement and breathing mechanics [3].

Clinically, the SCM is notable because of its anatomic relationships and its frequent involvement in musculoskeletal complaints such as cervicogenic headache, temporomandibular disorders, and postural dysfunctions [4,5,6,7]. Accordingly, prior work has examined its behavior using electromyography (EMG) to characterize activation patterns [8,9], evaluated pain responses [10], and quantified stiffness [9] and morphology [11,12].

Myofascial trigger points (MTrPs), which are defined as hyper-irritable, palpable nodules within taut bands that can provoke local and referred pain and reproduce patients’ symptomatic patterns when stimulated [13], are commonly found within the SCM muscle in conditions including chronic tension-type headache and other cervicogenic syndromes [14]. Because mechanical abnormalities may help sustain chronic pain [15], measuring SCM stiffness is a plausible marker of dysfunction. Notably, shear-wave elastography (SWE) studies report that individuals with chronic neck pain can show reduced SCM stiffness compared with healthy controls despite similar EMG activity, implying that stiffness may capture adaptations not evident on EMG or on structural B-mode ultrasound [9]. Beyond baseline characterization, documenting how stiffness changes after manual or instrument-assisted interventions is relevant for both clinicians and researchers.

Before drawing clinical inferences about SCM stiffness using SWE, it is essential to establish the technique’s measurement properties and normative values in a pain-free population. Because SWE is operator-dependent, beginning with healthy participants helps isolate instrument and procedural sources of variability (e.g., probe placement, transducer pressure, fiber orientation, cervical position, and respiratory phase) without confounding influences of pathology such as antalgic guarding, reactive hypertonia, or altered motor control that could inflate variability. This step enables protocol optimization (region of interest definition, number of acquisitions, and acceptable averaging), precise quantification of intra- and inter-examiner reliability, and calculation of error metrics (SEM and MDC) alongside sex- and side-specific reference ranges. Having these benchmarks is crucial to determine whether subsequent differences observed in patients represent true changes or differences in stiffness rather than measurement noise and to power both cross-sectional and longitudinal studies appropriately. Accordingly, the aim of this study is to present a reproducible SWE protocol to quantify SCM stiffness and to report intra- and inter-examiner reliability in healthy volunteers as a methodological foundation for future clinical research.

## 2. Materials and Methods

### 2.1. Study Design

Between 12 November and 21 November (2025), asymptomatic volunteers were recruited within the Physiotherapy Department of Francisco de Vitoria University (Madrid, Spain) to carry out a longitudinal observational study focused on intra- and inter-examiner reliability. Reporting followed the Guidelines for Reporting Reliability and Agreement Studies (GRRAS) to enhance methodological transparency and quality [16]. The study protocol was reviewed and approved prior to enrollment by the Ethics Committees of Rey Juan Carlos.

### 2.2. Eligibility Criteria

Volunteers without neck pain were recruited via convenience sampling after posting local notices throughout the faculty. Eligibility required ages 18–65, absence of current neck pain, and an asymptomatic status for at least the previous six months.

Exclusion criteria comprised any ongoing medical, pharmacological, or physiotherapy interventions in the prior six months that could alter pain perception or muscle tone; a history of traumatic cervical disorders (e.g., whiplash-associated disorders, fractures, and fissures); prior neck surgery; signs or history of radiculopathy or myelopathy; and systemic conditions such as fibromyalgia or oncologic disease. To reinforce asymptomatic classification, participants had to present a Neck Disability Index (NDI) ≤ 8/100 [17] and a Numeric Pain Rating Scale (NPRS) score ≤ 2/10 at rest and over the preceding week, which are thresholds commonly used to differentiate asymptomatic individuals from those with neck pain [18].

Written informed consent was obtained from all participants before any study procedures.

### 2.3. Sample Size Calculation

The minimum sample size was estimated using the approach described by Walter et al. [19], informed by intraclass correlation coefficients (ICCs) from earlier work assessing cervical muscles with SWE [20]. Assumptions were as follows: (1) a minimally acceptable ICC of 0.70, consistent with commonly cited thresholds for “good” reliability [21]; (2) an expected improvement from the ICC = 0.554 reported in a previous study [20] to ICC > 0.90, considering the methodological choices addressed in this study which could (hypothetically) improve the reference estimates; (3) 95% statistical power with a two-sided α = 0.05; and (4) an anticipated 10% attrition given the longitudinal design. Under these parameters, the required minimum number was 42 muscles for intra-examiner analyses (*n* = 42 SWE images in Trial 1 and *n* = 42 in Trial 2) and 42 muscles for inter-examiner analyses (*n* = 42 images from Examiner 1 and *n* = 42 from Examiner 2).

It should be noted that, although the increase from the reference ICC reported in prior work to a value around 0.90 may appear substantial, similar ICC improvements have been reported in cervical SWE reliability studies when acquisition conditions and region of interest (ROI) definition are highly standardized (e.g., landmark-based protocols with consistent ROI selection and stabilization prior to capture) and repeated acquisitions are averaged [22]. The authors of this study explicitly highlighted that earlier work by Bedewi et al. [23] was affected by limited procedural detail and a less reproducible anatomical reference, as participant positioning was only described as “supine” and probe placement was defined broadly as “beside the thyroid lobe,” which may introduce non-trivial variability in transducer location and ROI placement between sessions and examiners. In contrast, the anterior scalene protocol incorporated methodological refinements intended to reduce these sources of error, including a vertebral landmark-based approach to localize the target level (C7) using identifiable transverse process features, standardized console presets and relaxation instructions, and a clearly defined ROI strategy by contouring the muscle perimeter while avoiding inclusion of adjacent osseous, neural, and connective tissues. Using this more detailed and reproducible acquisition/ROI definition, the authors reported excellent test–retest reliability (ICC > 0.90) for both Young’s modulus and shear-wave speed when values were derived from averaged repeated acquisitions, supporting that reliability estimates can increase substantially when methodological variability is minimized.

Post hoc, using the final analyzed sample (*n* = 25 participants with bilateral SCM assessment, i.e., 50 muscles) and the same Walter et al. [19] framework (two measurements/raters, two-sided α = 0.05), the achieved power to detect an ICC of 0.90 against the minimally acceptable ICC of 0.70 was 99.5%, confirming that the study remained adequately powered for the planned reliability target. For transparency, if the observed inter-examiner ICC range (0.77–0.87) is used as the alternative ICC, the achieved power spans approximately 28% to 95%, reflecting the expected dependence of power on the true ICC value; therefore, we also emphasize the reported 95% confidence interval as the primary indicator of estimate precision.

### 2.4. Examiners

Two assessors with different experience levels acquired and analyzed all ultrasound data. The “experienced” examiner had >10 years of experience in musculoskeletal ultrasound and clinical assessment; the “novice” examiner had less than 20 h of training, 1 year of experience and no previous use of the same device.

Prior to data collection, both assessors completed a structured 2 h training session on the ultrasound device used for this study to standardize the full acquisition and analysis workflow. The session covered a review of the anatomical landmarks and participant positioning, console setup and parameter locking to ensure identical presets, probe handling drills (minimal transducer pressure, adequate gel, alignment parallel to fiber direction for longitudinal B-mode, and orthogonal insonation to minimize anisotropy), SWE-specific procedures (activating the mode, placing an ROI spanning the full muscle thickness while excluding fascia, maintaining a stable image for approximately 10 s before freezing, and recording both Young’s modulus and shear-wave velocity) and acceptance criteria for frames based on image/quality map stability and absence of motion or artifact. Each assessor practiced the full protocol on a pilot volunteer, performed three repeated measurements per site, and recorded values in the study datasheet to rehearse blinding and data entry conventions. Discrepancies were debriefed immediately, with corrective feedback and a checklist used to harmonize decisions on ROI placement and artifact rejection before commencing the study acquisitions.

Data collection was conducted in a dedicated, equipped ultrasound room with all required consumables. For each participant, the experienced examiner acquired one image per side; then, the novice repeated the procedure, after which the experienced examiner performed a second pass, followed by a final pass by the novice, yielding four acquisitions per side in immediate succession to minimize time-related variability. After each examiner, participants were asked to step off the table and walk briefly. The initial side assessed was randomized for each turn.

### 2.5. Shear-Wave Elastography Protocol for Measuring General Sternocleidomastoid Stiffness

**Figure 1:**
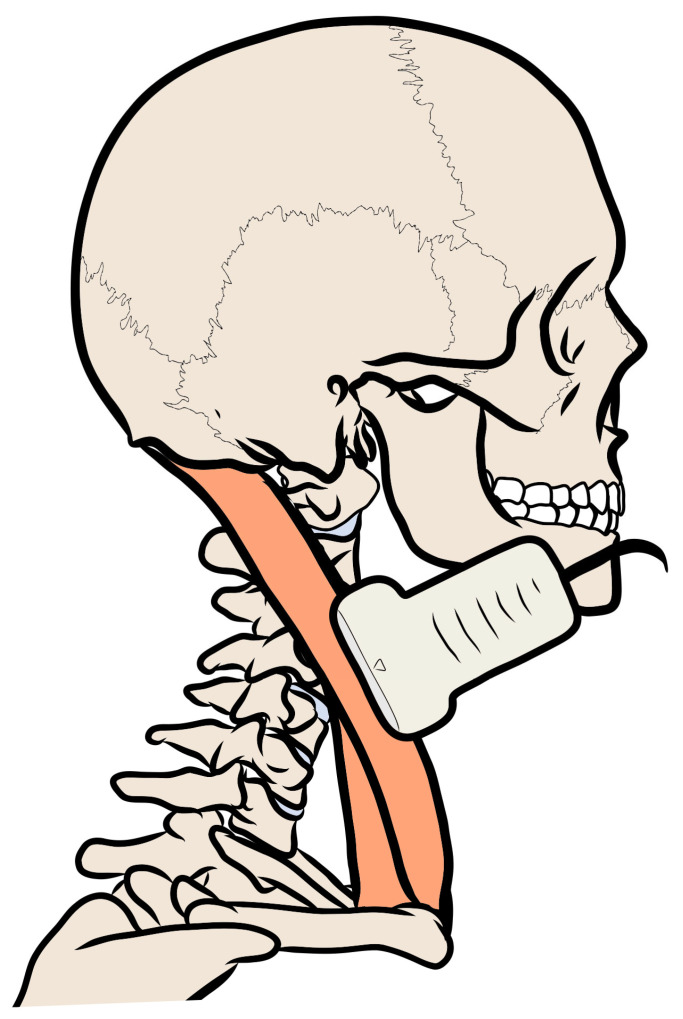
Schematic transducer positioning to visualize the sternocleidomastoid muscle.

All images were obtained on a LOGIQ E9 system with a 6–15 MHz linear transducer (ML-6-15-D; General Electric Healthcare, Milwaukee, WI, USA). Console parameters were standardized across acquisitions. Participants lay supine with a small pillow under their knees to reduce lumbar lordosis and were instructed to relax the cervical musculature to minimize contraction-related variability. The measurement site was standardized at the midpoint between the mastoid process and the sternal attachment of the SCM. With this point identified and marked, the probe was aligned parallel to the muscle fibers to capture a longitudinal view (Figure 1).

On B-mode imaging, the SCM was recognized as a relatively thick, superficial muscle beneath the subcutaneous tissue and platysma. Care was taken to align the muscle belly orthogonally with respect to the insonation plane to avoid artifacts that could bias stiffness estimates.

For SWE acquisition and analysis, the imaging mode was activated on console. SWE estimates tissue stiffness from the propagation of mechanically induced shear waves within the tissue. Briefly, focused acoustic radiation force (“push”) pulses generated by the transducer induce a small, localized displacement along the ultrasound beam. This displacement generates transverse shear waves that travel predominantly perpendicular to the beam and propagate laterally through the tissue. Ultrafast imaging then tracks the resulting micrometric displacements over time, enabling computation of the local shear-wave speed (SWS) within the selected ROI [24].

From a biomechanical standpoint, SWS is related to the shear modulus (G) of the tissue. Under the conventional assumptions of an isotropic, nearly incompressible medium with approximately constant density (ρ), shear-wave propagation follows (V = \sqrt{G/\rho}), such that (G = \rho \cdot V^{2}) [25]. Many ultrasound systems additionally report Young’s modulus (E) by applying a model-based conversion from SWS (commonly (E \approx 3\rho V^{2}) under the incompressible-isotropic approximation). In the present study, SWS (m/s) and Young’s modulus (kPa) were recorded as displayed by the ultrasound device; modulus values were therefore device-derived estimates based on the manufacturer’s default conversion assumptions, including tissue density ≈ 1000–1050 kg/m^3^ and Poisson’s ratio ≈ 0.5. These parameters were not measured in vivo and were not modified in the system settings. In practical terms, higher SWSs (and higher derived modulus values) correspond to greater tissue stiffness, whereas lower values indicate a more compliant muscle. SWE produces quantitative, spatially resolved stiffness maps that can be overlaid on B-mode images to support consistent ROI placement and is less dependent on operator-applied compression than strain-based techniques, which supports objective and reproducible stiffness measurements in muscles, including deeper structures [26,27].

**Figure 2:**
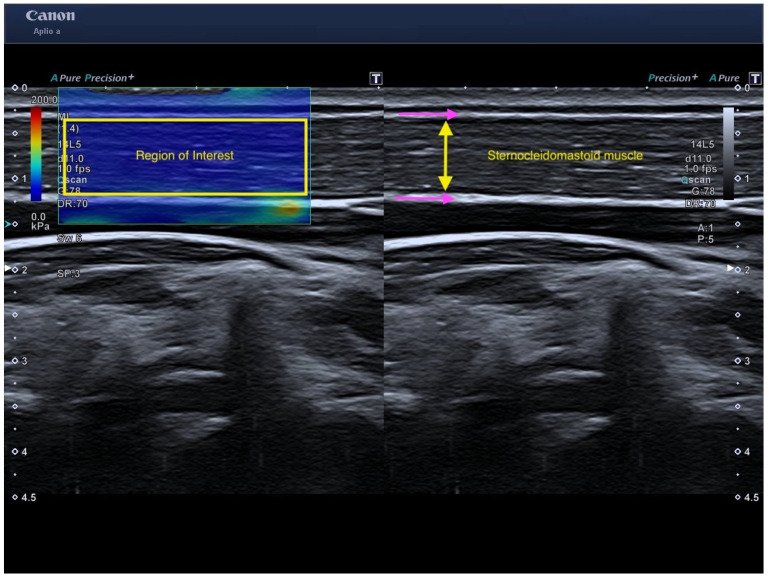
Representative shear-wave elastography image of the sternocleidomastoid muscle acquired under the standardized protocol. The region of interest spans the muscle belly and excludes fascia (pink arrows) (Canon: Milwaukee, WI, USA).

Therefore, the region of interest (ROI) was positioned to encompass the full muscle thickness (excluding surrounding fascia and other connective tissues) and at least 2 cm of fiber length. After stabilizing the ROI and waiting approximately 10 s, the frame was frozen, and the system automatically computed Young’s modulus and shear-wave velocity (Figure 2).

### 2.6. Statistical Analysis

Data processing was performed in SPSS for Mac (v.29; Armonk, NY, USA). All tests were two-tailed with α = 0.05. Distributional assumptions were inspected using histograms and Shapiro–Wilk tests (*p* > 0.05 indicating normality). Descriptive statistics summarized sample characteristics. Intra-examiner (test–retest) reliability was evaluated using the following: (1) the mean of both trials; (2) absolute error between trials; (3) ICC_3,1_ from a two-way mixed-effects, consistency model; (4) the standard error of measurement (SEM = SD of the trial mean × √(1 − ICC)); and (5) the minimal detectable change (MDC = SEM × 1.96 × √2) [21]. Inter-examiner reliability was calculated separately for single measurements and the mean of two measurements, reporting the grand mean across examiners, absolute inter-examiner error, ICC_3,2_ (two-way mixed-effects and consistency), SEM, and MDC [21].

## 3. Results

From the *n* = 26 participants initially recruited, *n* = 1 declined participation during data collection; thus, *n* = 25 participants (34.8% males) were included in the analysis. Since, for each participant, both sides were assessed (two sides), two examiners performed the measurements (two examiners), and each examiner completed two sessions (two sessions), yielding eight acquisitions per participant, and a total of 200 images were obtained (25 × 2 × 2 × 2). None of the images were discarded and, therefore, all were analyzed.

Participant characteristics are summarized in Table 1. Males were slightly older (*p* > 0.05) and had a significantly higher height (*p* < 0.001), weight (*p* < 0.001) and BMI (*p* = 0.019). Regarding tissue properties, none of the muscle stiffness metrics acquired with SWE showed significant differences between sides (in either men or women) or between sexes (all comparisons yielded *p* > 0.05).

Inter-examiner reliability analyses are summarized in Table 2. The data suggest that the agreement between the novice and experienced examiners was good–excellent. Single-measurement ICCs ranged from ~0.77 (SWS) to ~0.86 (shear modulus) and improved slightly when averaging two trials (~0.79–0.87), with corresponding reductions in SEM and MDC (shear modulus SEM/MDC ≈ 8.5/23.6 kPa → 7.4/20.5 kPa; shear-wave speed SEM/MDC ≈ 0.45/1.24 m/s → 0.40/1.10 m/s). Between-examiner mean differences were small and nonsignificant for both modulus and SWS (*p* ≥ 0.068), indicating no systematic bias. Practically, using the average of two acquisitions enhances precision and lowers the minimum detectable change, especially for shear modulus, while shear-wave speed shows consistently low absolute error.

Intra-examiner reliability results are reported in Table 3. Intra-rater reliability for sternocleidomastoid stiffness was excellent for both examiners and both metrics. ICC_3,1_ values clustered around 0.93–0.94 for Young’s modulus and shear-wave speed, with trivial trial-to-trial mean differences (all *p* ≥ 0.311), indicating no systematic bias. Absolute error was small (≈8 kPa and ≈0.3–0.4 m/s), and precision was high (SEM ≈ 5–6 kPa and 0.22 m/s), yielding MDCs of ~15–16 kPa and ~0.60 m/s.

## 4. Discussion

Nagai et al. [20] conducted the first study that, among other objectives, aimed to analyze intra-examiner reliability to calculate SCM muscle stiffness using SWE. The authors described that a single examiner acquired images twice, separated by a 20 min break. Although the selected location seemed to be highly reproducible (the midpoint between the mastoid process and the sternoclavicular joint orienting the transducer longitudinally along the fibers’ direction, similarly to our study), the ICC obtained was not good (ICC = 0.554). As the authors recognize, several reasons could explain these poor results. Post-processing by a single investigator with a variably sized and position circular ROI (likely altering values when smaller versus larger areas are sampled [28,29]), device software variability [30] (e.g., ROI geometry options or default assumptions such as Poisson’s ratio and tissue density used to convert shear-wave speed to shear modulus [27,31]), risk of type II error given the limited sample size (*n* = 13), and long sessions (~90 min) that could induce discomfort and inadvertent muscle activation were potential factors which could explain the moderate reliability.

Considering that many of these factors are modifiable, our study was designed to test whether methodological refinements (e.g., a larger sample size, shorter assessment sessions, using a different device, considering stricter standardization of posture and probe handling, defining a wide rectangular-shaped ROI spanning the full muscle thickness while excluding fascia and other noncontractile tissues, which reduces the risk of shifting values as the sampled circular area changes, a 10 s time of image stabilization, careful alignment of the muscle belly orthogonally with respect to the insonation plane to avoid artifacts and a comparison of acquiring single images or averaging two trials) could improve reproducibility. In addition, we extended the scope beyond intra-examiner agreement by including two examiners with different expertise levels to quantify inter-examiner reliability and assess whether the protocol performs consistently across users.

As a result, this study found that SCM stiffness can be measured with high reproducibility when the acquisition and analysis are tightly standardized. Inter-examiner agreement between the novice and experienced assessors was consistently good-to-excellent, with negligible between-examiner bias and further gains in precision when two acquisitions were averaged. On the other hand, intra-examiner repeatability was excellent for both shear modulus and shear-wave speed, with small trial-to-trial error and no systematic shifts between sessions.

Drawing on these results, we offer four pragmatic recommendations for SWE assessment of the SCM. First, recognize that methodological decisions are decisive for reproducibility. Rigorously standardized patient positioning, transducer alignment, an ROI that spans the full muscle thickness while excluding fascia, the stabilization time before image capture, and fixed console presets yielded excellent intra-examiner ICCs and reduced SEM/MDC to clinically manageable ranges, highlighting that protocol design is a critical condition to ensure reliability. Notably, the novice examiner achieved excellent intra-examiner reliability after a brief, structured training session, suggesting that (under standardized acquisition conditions) reliable SCM SWE measurements may be feasible even for less experienced operators, potentially broadening the technique’s applicability. Second, to avoid attributing apparent stiffness “changes or differences” to examiner-specific technique, ensure that the same operator acquires and interprets images across research visits and clinical follow-ups. Using a single operator preserved the excellent test–retest agreement and prevented cross-operator technique drift that can mimic physiological change. Third, if multiple examiners are unavoidable (due to specific research or clinical situations), average at least two acquisitions per side to dampen examiner-specific noise. Our results showed that this improved inter-examiner ICCs and lowered SEM/MDC, although the modest precision gain should be weighed against additional scan time and workflow costs to decide whether two-measurement averaging is warranted. Finally, for longitudinal assessments, prioritize experienced operators whenever possible. Despite similarly high intra-examiner ICCs, experienced examiners showed slightly lower MDCs, increasing the likelihood that observed changes in SCM stiffness reflect true physiological evolution rather than measurement error (crucial when monitoring patient progress or intervention effects over time).

### Limitations

This study has several limitations. First, the sample comprised asymptomatic young adults recruited by convenience, with an imbalanced sex distribution, which limits generalizability (in terms of normative or reference values) to clinical populations with neck pain or to older cohorts. In addition, tissue alterations in symptomatic groups [12,31] may hinder fascial boundary definition and affect reliability. Second, measurements were restricted to a single standardized site in the supine, relaxed condition. Results may not extrapolate to other cervical levels, alternative positions, or contraction tasks. Third, acquisitions were obtained on one ultrasound platform using vendor-specific SWE implementations and a predefined rectangular ROI. Hardware/firmware, presets, and ROI geometry differences across devices could yield different reliability profiles, and we did not verify cross-device equivalence or phantom-based calibration. Fourth, the protocol emphasized short-term repeatability within a session. Day-to-day test–retest reliability, long-term drift, and inter-session MDCs were not assessed. Finally, converting shear-wave speed to Young’s modulus relies on assumptions (e.g., tissue density and Poisson’s ratio) that were not directly verified and may introduce model-based errors.

## 5. Conclusions

The results of this study support that a tightly standardized SWE protocol for the sternocleidomastoid is excellently repeatable if two measurements are acquired by the same examiner and good-to-excellent for two examiners (even if they have different levels of expertise), with negligible systematic bias and small measurement errors. Averaging two acquisitions improved the precision modestly, particularly between examiners, while single-operator assessments preserved the highest test–retest stability. Compared with prior reports of moderate reliability, these findings indicate that methodological refinements (standardized positioning and presets, orthogonal insonation, and a rectangular ROI spanning full muscle thickness while excluding fascia) materially enhance reproducibility. Therefore, our recommendations to ensure good reliability and measurement precision are (1) following the methodological considerations analyzed in this study, (2) maintaining a single operator for longitudinal monitoring when feasible, (3) averaging at least two acquisitions per side when multiple examiners are involved, and (4) prioritizing preference for experienced operators in follow-up contexts to minimize MDCs.

## Figures and Tables

**Table 1:** Descriptive analyses of demographic and clinical characteristics of the sample.

Variables	Sample (*n* = 25)	Males (*n* = 8)	Females (*n* = 17)	Gender Difference (95% Confidence Interval)
Demographics				
Age, years	21.7 ± 8.2	24.6 ± 10.5	20.4 ± 6.8	4.3 (−2.9; 11.4) *p* = 0.231
Height, m	1.69 ± 0.09	1.79 ± 0.06	1.65 ± 0.06	0.13 (0.07; 0.19) *p* < 0.001
Weight, kg	63.4 ± 12.9	75.6 ± 10.2	57.6 ± 9.7	18.1 (9.3; 26.8) *p* < 0.001
BMI, kg/m^2^	21.7 ± 2.8	23.5 ± 2.7	20.8 ± 2.5	2.7 (0.48; 4.97) *p* = 0.019
Shear modulus, kPa *				
Mean	45.1 ± 17.6	49.0 ± 22.9	43.2 ± 14.9	5.2 (−6.7; 17.0) *p* = 0.383
Right side	49.0 ± 22.9	56.0 ± 30.5	45.7 ± 18.5	
Left side	43.3 ± 15.0	43.3 ± 15.4	43.3 ± 15.3	
Difference	5.7 (−5.3; 16.1) *p* = 0.270	12.7 (−6.8; 32.3) *p* = 0.196	2.4 (−11.0; 15.8) *p* = 0.720	
Shear-wave speed, m/s *				
Mean	3.73 ± 0.75	3.75 ± 0.85	3.78 ± 0.79	0.24 (−0.531; 0.45) *p* = 0.876
Right side	3.89 ± 0.90	3.88 ± 1.14	3.90 ± 0.80	
Left side	3.65 ± 0.69	3.61 ± 0.85	3.68 ± 0.79	
Difference	0.23 (−0.21; 0.69) *p* = 0.302	0.26 (−0.66; 1.20) *p* = 0.549	0.22 (−0.33; 0.78) *p* = 0.429	

* Scores are calculated based on the mean average of both trials and examiners.

**Table 2:** Inter-examiner reliability analyses to determine general sternocleidomastoid muscle stiffness.

Reliability Estimates	Shear Modulus (kPa)		Shear-Wave Speed (m/s)	
	Novice Examiner	Experienced Examiner	Novice Examiner	Experienced Examiner
Single Measurements				
Mean	50.1 ± 22.8	44.6 ± 22.3	4.00 ± 0.93	3.65 ± 0.94
Difference	5.4 (−3.5; 14.4) *p* = 0.229		0.34 (−0.02; 0.71) *p* = 0.068	
Absolute Error	12.3 ± 11.3		0.61 ± 0.61	
ICC_3,2_, 0–1	0.858 (0.749; 0.919)		0.770 (0.594; 0.869)	
SEM	8.5		0.45	
MDC	23.6		1.24	
Mean Average of 2 Measurements				
Mean	48.4 ± 21.4	43.9 ± 19.8	3.90 ± 0.86	3.64 ± 0.89
Difference	4.5 (−3.6; 12.7) *p* = 0.276		0.26 (−0.08; 0.61)	
Absolute Error	11.1 ± 9.4		0.55 ± 0.54	
ICC_3,2_, 0–1	0.870 (0.771; 0.926)		0.791 (0.632; 0.882)	
SEM	7.4		0.40	
MDC	20.5		1.10	

ICC: Intraclass correlation coefficient; MDC: minimal detectable change; SEM: standard error of measurement.

**Table 3:** Test–retest reliability analyses to determine general sternocleidomastoid muscle stiffness.

Reliability Estimates	Novice Examiner				Experienced Examiner			
	Young’s Modulus (kPa)		Shear-Wave Speed (m/s)		Young’s Modulus (kPa)		Shear-Wave Speed (m/s)	
	Trial 1	Trial 2	Trial 1	Trial 2	Trial 1	Trial 2	Trial 1	Trial 2
Mean	50.1 ± 22.8	46.7 ± 21.5	4.00 ± 0.93	3.81 ± 0.86	44.6 ± 22.3	43.1 ± 18.5	3.65 ± 0.94	3.63 ± 0.90
Difference	3.4 (−5.4; 12.2) *p* = 0.447		0.18 (−0.17; 0.53) *p* = 0.311		1.5 (−6.6; 9.6) *p* = 0.717		0.02 (−0.34; 0.38) *p* = 0.914	
Absolute Error	8.1 ± 8.1		0.37 ± 0.30		7.2 ± 7.0		0.32 ± 0.31	
ICC_3,1_, 0–1	0.930 (0.879; 0.960)		0.934 (0.883; 0.962)		0.929 (0.875; 0.960)		0.936 (0.887; 0.964)	
SEM	5.7		5.3		0.22		0.22	
MDC	15.8		0.60		14.7		0.60	

ICC: Intraclass correlation coefficient; MDC: minimal detectable change; SEM: standard error of measurement.

## Data Availability

The datasets used and/or analyzed during the current study are available from the corresponding author on reasonable request.
